# Acknowledgement of manuscript reviewers 2014

**DOI:** 10.1186/s13031-015-0033-x

**Published:** 2015-02-06

**Authors:** Olivier Degomme, Ruwan Ratnayake, Bayard Roberts, Ines Keygnaert, Adrianna Murphy, An-Sofie Van Parys

**Affiliations:** Ghent University, Brussels, Belgium; International Rescue Committee, Washington, United States of America; London School of Hygiene and Tropical Medicine, London, United Kingdom

## Abstract

The editors of *Conflict and Health* would like to thank all our reviewers who have contributed to the journal in Volume 8 (2014).

**Hirotsugu Aiga**

Japan

**Neil Arya**

Canada

**Karl Blanchet**

United Kingdom

**Marleen Bosmans**

Belgium

**Moriah Brier**

United States of America

**Frederick Burkle**

United States of America

**Alessandro Cassini**

Sweden

**Sandro Colombo**

Spain

**Stephen Commins**

United States of America

**Susan Cookson**

United States of America

**Bart Criel**

Belgium

**Claude de Ville de Goyet**

Belgium

**Jocelyn DeJong**

Lebanon

**Pamela DeLargy**

United States of America

**Wim Delva**

South Africa

**Delan Devakumar**

United Kingdom

**Lesley Doyal**

United Kingdom

**Anbrasi Edward**

United States of America

**Jack Eldon**

United Kingdom

**Helen Elsey**

United Kingdom

**Jeroen Ensink**

United Kingdom

**Larissa Fast**

United States of America

**Nathan Ford**

South Africa

**Noriko Fujita**

Japan

**Ananda Galappatti**

Sri Lanka

**Richard Garfield**

United States of America

**Asha George**

United States of America

**Gregg Greenough**

United States of America

**Andre Griekspoor**

Switzerland

**Tanya Guenther**

United States of America

**Debby Guha-Sapir**

Belgium

**Peter Hill**

Australia

**Lara Ho**

Switzerland

**Keng-Yen Huang**

United States of America

**Michelle Hynes**

United States of America

**Paul Ickx**

Belgium

**Sumit Kane**

Netherlands

**Unni Krishnan Karunakara**

India

**Stephanie Kayden**

United States of America

**Erin Kenny**

United States of America

**Albert Kilian**

Spain

**Louis Lillywhite**

United Kingdom

**Inas Mahdi**

United States of America

**Suman Majumdar**

Australia

**Robert Marten**

United States of America

**Tim Martineau**

United Kingdom

**Farrah Mateen**

United States of America

**Verena Mauch**

Germany

**Barbara McPake**

United Kingdom

**William Moss**

United States of America

**Adrianna Murphy**

United Kingdom

**Robin Nandy**

Indonesia

**Sonia Navani**

United States of America

**Colleen O'Connell**

Canada

**Emilomo Ogbe**

Belgium

**Donal O'Mathuna**

Ireland

**Christopher Orach**

Uganda

**Kathleen O'Reilly**

United Kingdom

**Jennifer Palmer**

United Kingdom

**Preeti Patel**

United Kingdom

**Enrico Pavignani**

Mozambique

**Duncan Pedersen**

Canada

**Valerie Percival**

Canada

**Catherine Pitt**

United Kingdom

**Anita Ramesh**

United Kingdom

**Elizabeth Reed**

United States of America

**Amelia Reese Masterson**

United States of America

**Kristien Roelens**

Belgium

**Simon Rushton**

United Kingdom

**Khwaja Mir Islam Saeed**

Afghanistan

**Ouahiba Sakani**

Algeria

**Abdul Tawab Saljuqi**

United States of America

**Jennifer Scott**

United States of America

**Simukai Shamu**

South Africa

**Sudesh Raj Sharma**

Nepal

**Chesmal Siriwardhana**

Sri Lanka

**Joyce Smith**

Indonesia

**Sarah Smith**

United Kingdom

**Daya Somasundaram**

Sri Lanka

**Egbert Sondorp**

United Kingdom

**Paul Spiegel**

Switzerland

**Eliana Suarez**

Canada

**May Sudhinaraset**

United States of America

**Nathan Taback**

Canada

**Christine Kirunga Tashobya**

Uganda

**Annemarie ter Veen**

Netherlands

**Vianney Tricou**

Central African Republic

**Margaret Usher-Patel**

United Kingdom

**Tine Van Bortel**

United Kingdom

**Wim Van Damme**

Belgium

**Edwin van Teijlingen**

United Kingdom

**Patrick Van de Voorde**

Belgium

**Peter Ventevogel**

Netherlands

**Lena Verdeli**

United States of America

**Anna Whelan**

Australia

**Ashley Wolfington**

United States of America

